# Development and Characterization of Wheat Burger Buns Fortified with Fermented–Freeze-Dried Sesame Bran Powder: Nutritional and Sensory Evaluation

**DOI:** 10.3390/foods15183191

**Published:** 2026-09-09

**Authors:** Rehab F. M. Ali, Ayman M. El-Anany, Raed Alayouni

**Affiliations:** 1 Department of Food Science and Human Nutrition, College of Agriculture and Food, Qassim University, Buraydah 51452, Saudi Arabia; r.alayouni@qu.edu.sa; 2 Special Food and Nutrition Department, Food Technology Research Institute, Agricultural Research Center, Giza 12619, Egypt; aymanelanany82@gmail.com

**Keywords:** diet, antioxidant activity, fortification, fermented sesame bran powder, wheat burger buns, nutritional quality, sensory evaluation, functional food

## Abstract

Conventional wheat flour burger buns are often energy-dense but deficient in essential nutrients. This study investigated their fortification with Fermented–Freeze-Dried Sesame Bran Powder (FFSBP), a sustainable, nutrient-rich by-product. FFSBP was produced via solid-state fermentation using *Lactobacillus plantarum*, followed by freeze-drying, and incorporated into refined wheat flour at 0% (control), 1%, 2%, 3%, 4%, and 5% (*w*/*w*). Proximate composition, functional properties, physical characteristics, instrumental color, bioactive content, antioxidant activity, and sensory attributes were evaluated. FFSBP significantly (*p* < 0.05) and dose-dependently improved nutritional quality, increasing crude protein to 13.92% (a relative increase of approximately 9% compared with the control), dietary fiber by 238% (from 0.76% to 2.57%), ash mineral content, and total phenolic/flavonoid compounds. Antioxidant activity, measured via DPPH radical scavenging, increased nearly fourfold. Instrumental color analysis revealed dose-dependent darkening of the crust and crumb, with decreased lightness (L) and increased yellowness (b). However, specific volume decreased by approximately 7% at the 5% level. Sensory evaluation showed that buns with up to 3% FFSBP maintained overall acceptability comparable to the control, benefiting from enhanced taste and odor, whereas 4–5% levels negatively impacted appearance, crumb color, and mouthfeel texture, correlating with color shifts. The results demonstrate that 3% FFSBP optimally enhances nutritional and bioactive properties without compromising sensory acceptance or physical structure.

## 1. Introduction

Bakery products form a fundamental component of diets worldwide, with staple items such as buns and bread being consumed daily by billions of people. Their pervasive consumption provides an efficient and passive means for public health nutrition intervention through targeted fortification [1,2,3]. This opportunity is particularly salient because many conventional bakery products rely heavily on refined wheat flour, which is often energy-dense but deficient in essential nutrients including dietary fiber, high-quality protein, and bioactive phytochemicals [4,5]. In response, fortification, defined as the deliberate incorporation of functional ingredients, has emerged as a critical strategy to mitigate nutritional inadequacies and convert everyday foods into vehicles for health-promoting compounds [6]. The enrichment of bakery products with varied sources of fiber, protein, and bioactive constituents represents more than a passing trend; it is an essential progression in food science aimed at improving dietary quality [7]. This approach directly addresses widespread nutritional shortfalls linked to modern eating patterns, such as insufficient fiber and suboptimal protein intake [8]. The integration of ingredients like legume flours, oilseed meals, fruit pomaces, and cereal brans can markedly improve the nutritional profile of common products like burger and breakfast buns [8,9]. Specifically, fiber fortification promotes digestive health and satiety, protein enhancement supports muscular and metabolic functions, and bioactive components such as polyphenols, antioxidants, and lignans help mitigate oxidative stress and inflammation [10,11]. Concurrently, increasing consumer demand for healthier and more sustainable food options has spurred interest in nutrient-rich, functional ingredients derived from underutilized agro-industrial by-products [12,13]. Sesame bran powder, a by-product of sesame oil extraction (*Sesamum indicum* L.), is one such material with considerable nutritional value yet remains largely underexploited [14]. Produced in significant quantities as a residue of the global sesame oil industry [15], sesame bran has historically been relegated to low-value applications such as animal feed or fertilizer. However, recent research has highlighted its potential as a sustainable, functionally rich food ingredient [16]. Its composition features a concentrated array of bioactive compounds and essential nutrients retained after oil processing. Notably, sesame bran is an excellent source of high-quality plant protein with a balanced amino acid profile, a rich reservoir of dietary fiber (especially insoluble fiber), and a significant carrier of unique lignans like sesamin and sesamolin, which are associated with antioxidant, anti-inflammatory, and hypocholesterolemic properties [17,18]. Additionally, it contains appreciable levels of minerals including calcium, iron, magnesium, and zinc, as well as B-vitamins [19]. The application of microbial fermentation, particularly using lactic acid bacteria (LAB), has been established as an effective method to modify the chemical and structural matrix of bran components [20,21]. This bioprocess actively enriches the bran through several mechanisms: (i) the enzymatic degradation of complex polysaccharides, which increases soluble dietary fiber content and improves prebiotic potential; (ii) the hydrolysis of proteins into bioactive peptides and free amino acids, enhancing digestibility and nutritional value; (iii) the liberation of bound phenolic compounds from cell wall structures, increasing their extractability and antioxidant capacity; and (iv) the reduction in antinutritional factors such as phytic acid, which improves mineral bioavailability [22,23,24,25]. Furthermore, fermentation generates organic acids and other metabolites that contribute to improved flavor profiles and natural preservation [24]. The selection of Lactobacillus plantarum for this study was based on its well-established metabolic versatility and proven efficacy in fermenting plant-based substrates. *L. plantarum* is a widely used starter culture in food fermentation, known for its ability to produce a diverse array of enzymes, including phytases, proteases, and glycosidases, which facilitate the biotransformation of bran components [20]. This species has been successfully employed in the fermentation of various cereal brans, including wheat, rice, and oat brans, demonstrating consistent improvements in nutritional quality, antioxidant activity, and functional properties [21]. Its Generally Recognized as Safe (GRAS) status and robust growth in solid-state fermentation systems further support its suitability for food applications. Previous studies have successfully incorporated fermented by-products into bakery products. For instance, fermented wheat bran has been used to improve bread quality by enhancing dough rheology, increasing loaf volume, and improving sensory acceptance due to the production of flavor-active compounds during fermentation [26,27]. Similarly, fermented rice bran has been incorporated into baked goods to increase protein digestibility and antioxidant activity [20]. The fermentation of oilseed by-products, including sesame meal, has been shown to reduce antinutritional factors and improve protein quality [28]. These precedents demonstrate that fermentation not only valorizes agro-industrial by-products but also actively contributes to the technological and sensory properties of the final baked products, making them more acceptable to consumers. Therefore, the main objective of this study is to comprehensively evaluate the nutritional composition, functional properties, bioactive content, antioxidant activity, physical characteristics, color attributes, and sensory characteristics of wheat flour burger buns incorporated with different quantities of Fermented–Freeze-Dried Sesame Bran Powder (FFSBP).

## 2. Materials and Methods

### 2.1. Raw Material and Chemicals

The sesame bran powder (SBP) was sourced as an industrial by-product from tahini production at a processing facility in Unizah, Qassim, Saudi Arabia. The SBP was collected fresh from the production line, immediately sealed in food-grade polyethylene bags, and transported to the laboratory within 2 h under refrigerated conditions (4 °C). Upon arrival, it was stored at −20 °C until further processing to prevent lipid oxidation and microbial spoilage. Superior Wheat Flour with 70% extraction was sourced from the Kuwait Flour Mills & Bakeries Company in Kuwait City, Kuwait. The flour had a crude protein content of 11.70% and a farinograph water absorption of 60.0% at 500 FU, indicating medium dough strength suitable for bun production. The remaining ingredients, including Bakemate instant dry yeast (*Saccharomyces cerevisiae*), Bread improver (Ireks champion, İREKS GIDA SANAYİ A.Ş., Istanbul, Turkey; composition: wheat flour, microbial alpha-amylase, ascorbic acid, calcium carbonate, diacetyl tartaric acid esters of mono and diglycerides), sunflower oil, white sugar, full cream milk powder and salt were procured from a local market from a local market in Buraydah, Qassim, Saudi Arabia. *Lactobacillus plantarum* EM. was procured from MIRCEN (Microbial Resource Centre), Ain Shams University, Egypt. All chemicals used were of analytical grade and were purchased from Sigma-Aldrich (St. Louis, MO, USA) and Merck (Darmstadt, Germany), unless otherwise specified. Food-grade ingredients (yeast, bread improver, oil, sugar, milk powder, salt) were of commercial food quality.

### 2.2. Preparation of Fermented–Freeze-Dried Sesame Bran Powder

SBP was subjected to a solid-state fermentation process using *Lactobacillus plantarum* EM. A stock culture of *L. plantarum* EM (MRS agar slant) was reactivated by transferring a loopful into 10 mL of sterile de Man, Rogosa and Sharpe (MRS) broth and incubating at 37 °C for 18 h. This pre-culture was then used to inoculate 100 mL of fresh MRS broth at an inoculum size of 2% (*v*/*v*) and incubated under the same conditions to obtain an active starter culture [20]. For fermentation, a 10% (*w*/*v*) suspension of sesame bran powder in sterile distilled water was prepared in autoclavable flasks. The suspension was sterilized by autoclaving at 121 °C for 15 min. After cooling to room temperature, the sterilized substrate was inoculated with the active *L. plantarum* EM culture at an initial cell density of approximately 10^7^ CFU/mL. Fermentation was carried out under static conditions at 37 °C for 24 h. The initial pH of the substrate was adjusted to 6.0 ± 0.2 using 1 M NaOH or HCl. The pH was monitored at 0, 6, 12, and 24 h using a calibrated pH meter (Mettler Toledo, Greifensee, Switzerland) to ensure optimal microbial activity. The pH decreased progressively from 6.0 to 3.8 ± 0.1 over the 24 h fermentation period, indicating successful acid production by *L. plantarum* [20]. A non-inoculated, sterilized sesame bran suspension served as the unfermented control. Following the 24 h incubation, the microorganisms were inactivated by heating at 80 °C for 10 min in a water bath to halt microbial activity [20]. The resultant slurry was then freeze-dried using a Labconco Freeze Dryer Freezone 2.5 (Labconco, Kansas City, MO, USA). The freeze-drying conditions were as follows: condenser temperature of −50 °C, shelf temperature of 25 °C, and vacuum pressure of 0.05 mBar. The drying process was continued for 48 h until the moisture content was reduced to below 7% (*w*/*w*). The dried material was milled into a fine powder using an electric grinder (Braun Model 1021, Kronberg im Taunus, Germany), passed through a 150-mesh sieve, and stored in airtight containers at 4 °C until further use in bun formulation.

### 2.3. Preparation of Wheat Flour FFSBP Composite Flours

Fermented–Freeze-Dried Sesame Bran Powder (FFSBP) was incorporated into refined wheat flour via partial substitution at six distinct levels: 0% (control), 1%, 2%, 3%, 4%, and 5% (*w*/*w*). These specific substitution levels were selected based on preliminary formulation and baking assessments that evaluated the impact of FFSBP on dough handling properties, baking performance, and sensory acceptability. The range of 1–5% was chosen to identify the maximum incorporation level that could provide significant nutritional enhancement while maintaining acceptable physical and sensory characteristics. Preliminary studies indicated that levels above 5% resulted in excessive dough stickiness, poor gas retention, and unacceptable textural properties in the final product. To achieve a homogeneous blend, the wheat flour and FFSBP were dry-mixed using a Black+Decker kitchen stand dough mixer for 15 min. The resulting composite flours were immediately transferred to airtight polypropylene bags and stored under refrigeration at 4 ± 1 °C until use, a period not exceeding 48 h.

### 2.4. Wheat Burger Buns Formulation and Baking Procedure

The formulation of control and fortified doughs is detailed in Table 1. Bun production followed the straight-dough method [3]. Water addition for each composite flour was standardized based on its farinograph water absorption at 500 FU (Table 1). In a stand mixer, the composite flour, bread improver, instant dry yeast, sugar, and salt were dry-mixed for one minute. Half of the specified vegetable oil was then added, and mixing continued at low speed for 5 min. Subsequently, the remaining half of the oil was incorporated, and the dough was mixed at high speed for 4 min to achieve full gluten development. The developed dough was rested for 5 min at room temperature. After resting, the dough was divided and scaled into 80 g balls. The dough pieces were placed on greased baking pans and subjected to proofing in a fermentation cabinet at 38 °C and 85% relative humidity for 60 min. Proofed buns were baked in a preheated deck oven (Real Forni, Model BE 4C12.30, Gazzolo d’Arcole, Italy) at 220 °C for 11 min. Upon removal from the oven, the buns were cooled for 2 h in a controlled cooling chamber maintained at 25 °C. After the full cooling period (3 h post-baking), the buns were packaged in food-grade polyethylene bags. These samples were then subjected to sensory evaluation and specific volume measurement.

### 2.5. Proximate Composition Analysis

The proximate composition was determined according to the official methods of the Association of Official Analytical Chemists [27]. Moisture content was quantified via the air-oven drying method (AOAC 925.09B). Crude protein content (N × 5.7) was measured using the Micro-Kjeldahl technique (AOAC 960.52). The total lipid (fat) content was determined by Soxhlet extraction with petroleum ether (AOAC 950.36). Crude fiber content was analyzed using a sequential acid and alkaline digestion system (AOAC 950.37). Total ash content was measured by incineration in a muffle furnace at 600 °C (AOAC 930.22). All proximate composition data are expressed on a dry weight basis. The total carbohydrate content was calculated by difference using the following formula: Total Carbohydrates (%) = 100 − (% Protein + % Fat + % Ash + % Crude Fiber). The moisture content is reported separately as a complementary parameter and is not included in the carbohydrate calculation. This approach is consistent with standard methodology [3]. The energy value (kcal/100 g) was subsequently calculated using the modified Atwater factors, as follows:

Energy (kcal/100 g) = (9 × % Fat) + (4 × % Protein) + (2 × % Crude Fiber) + (4 × % Total Carbohydrates)

### 2.6. Functional Properties Analysis

The functional properties, including water absorption capacity (WAC), oil absorption capacity (OAC), emulsion activity (EA), and foam capacity (FC), were evaluated for all flour samples using standard techniques [28,29,30]. For WAC and OAC, one-gram samples were each mixed with 10 mL of distilled water or refined soybean oil, respectively, held at 30 ± 2 °C for 30 min, and centrifuged at 2000× *g* for 30 min. The capacities were calculated as the percentage of water or oil bound per gram of flour. Emulsion activity was determined by homogenizing 1 g of flour with 10 mL of water and 10 mL of oil, followed by centrifugation at 2000× *g* for 5 min. EA was expressed as the percentage ratio of the emulsified layer height to the total mixture height. Foam capacity was assessed by vigorously shaking a suspension of 1 g flour in 50 mL of distilled water for 5 min. The foam volume measured 30 s after whipping was used to calculate FC percentage based on the volume increase.

### 2.7. Specific Volume of the Baked Burger Buns

The specific volume of the baked burger buns was determined according to the method described by Greene and Bovell-Benjamin [31]. The loaf volume (LV, cm^3^) was measured using the rapeseed displacement method in a graded container. The weight of each loaf was recorded using a sensitive electronic digital balance. The specific volume (SV, cm^3^/g) was then calculated as the ratio of the loaf volume to its weight (SV = LV/weight).

### 2.8. Bioactive Compounds and Antioxidant Activity Analysis

The bioactive profile and antioxidant potential of the sample extracts were characterized using established spectrophotometric methods. Extracts for analysis were prepared by mixing 1 g of each sample (ground burger bun) with 10 mL of 80% aqueous methanol. The mixture was sonicated for 30 min at room temperature, then centrifuged at 5000× *g* for 15 min. The supernatant was collected and used for subsequent analyses. The total phenolic content (TPC) was determined via the Folin–Ciocalteu assay [32]. Briefly, 0.5 mL of the extract was mixed with 2.5 mL of 10-fold diluted Folin–Ciocalteu reagent and incubated for 5 min at room temperature. Then, 2.0 mL of 7.5% sodium carbonate solution was added, and the mixture was incubated for 2 h at room temperature in the dark. Absorbance was measured at 765 nm using a UV-Vis spectrophotometer (Shimadzu UV-1800, Kyoto, Japan). Results were quantified against a gallic acid standard curve and are expressed as milligrams of gallic acid equivalents per gram of sample (mg GAE/g). The total flavonoid content (TFC) was measured using the aluminum chloride colorimetric method [33] with the following conditions: 0.5 mL of extract was mixed with 0.5 mL of 10% aluminum chloride, 0.5 mL of 1 M potassium acetate, and 4.5 mL of distilled water. The mixture was incubated for 40 min at room temperature, and absorbance was measured at 415 nm, with results reported as milligrams of catechin equivalents per gram (mg CE/g). Antioxidant capacity was assessed by evaluating the scavenging activity against the stable 1,1-diphenyl-2-picrylhydrazyl (DPPH) radical [32]. Specifically, 0.5 mL of extract was mixed with 2.5 mL of 0.1 mM DPPH solution in methanol. The mixture was incubated for 30 min in the dark at room temperature, and absorbance was measured at 517 nm. The percentage inhibition was calculated based on the reduction in absorbance at 517 nm relative to a control. All measurements were performed in five replicates.

### 2.9. Sensory Evaluation

**Ethics statement:** The study protocol for sensory evaluation was reviewed and approved by the Sensory Evaluation Ethics Committee, Food Technology Research Institute, Agricultural Research Center, Egypt (Approval No. SEC-40/2026). All panelists provided written informed consent prior to participation. A consumer panel of 65 untrained individuals (30 females, 35 males, aged 19–55 years) evaluated the burger bun samples for acceptability using a 9-point hedonic scale [34], where 1 indicated “extreme detest” and 9 indicated “extreme preference.” Samples (10 g) were served on coded, disposable trays in a controlled environment at 25 °C under daylight-equivalent fluorescent lighting. Panelists were instructed to cleanse their palates with water at 20 °C between samples. Evaluated sensory attributes included appearance, crumb color, odor, taste, mouthfeel texture, and overall acceptability.

### 2.10. Color Measurement

Color parameters (L, a, b*) were measured using a Konica Minolta colorimeter (Model CR-300, Konica Minolta, Osaka, Japan) calibrated with a white tile under D65 illumination. For crust color measurement, the entire top surface of each bun was placed directly under the colorimeter aperture, and five readings were taken from different positions on the crust surface. For crumb color measurement, each bun was sliced transversely (approximately 15 mm thickness), and the freshly exposed cut surface was placed under the colorimeter aperture, with five readings taken from different positions on the crumb surface. Samples were placed in Petri dishes to ensure uniform surface contact and eliminate air pockets. Five replicate readings per sample were taken for each attribute (crust and crumb) with the optical lens in contact with the dish lid under controlled ambient conditions.

### 2.11. Statistical Analysis

All experimental data, except for sensory results, were analyzed with five replicates (n = 5). Sensory data were analyzed based on responses from 65 panelists (n = 65). Statistical analyses were conducted using SPSS software (version 21.0, IBM Corp., Chicago, IL, USA). A one-way analysis of variance (ANOVA) was performed to determine significant differences among treatment means. Where ANOVA indicated significant effects (*p* < 0.05), means were separated using Duncan’s New Multiple Range Test, as outlined by Gomez and Gomez [35].

## 3. Results & Discussion

### 3.1. Proximate Composition of Superior Wheat Flour (SWF) and Fermented–Freeze-Dried Sesame Bran Powder (FFSBP) (g per 100 g Dry Weight Basis)

Table 2 shows the proximate composition of Superior Wheat Flour (SWF) and Fermented–Freeze-Dried Sesame Bran Powder (FFSBP) (g per 100 g dry weight basis). The proximate analysis revealed the foundational nutritional role of refined wheat flour as a high-energy, carbohydrate-dense matrix. Its composition of 86.35% total carbohydrates, yielding a high energy value of ~402 kcal/100 g, aligns with its established characterization as a staple food ingredient, primarily valued for its high starch content [36,37]. However, the refining process that yields this flour removes much of the bran and germ, resulting in a product that is notably low in crude fiber, crude protein, and ash (minerals) [36,37]. Specifically, this leads to low protein levels with deficiencies in essential amino acids [38] and a reduced mineral content [39]. This compositional profile contrasts significantly with the nutrient-dense profile of the FFSBP, which contained only 25.65% carbohydrates and a consequent lower energy value of ~313 kcal/100 g.

**Table 2:** Proximate composition of Superior Wheat Flour (SWF) and Fermented–Freeze-Dried Sesame Bran Powder (FFSBP).

Components (g/100 g Dry Weight Basis)	Superior Wheat Flour (SWF)	Fermented–Freeze-Dried Sesame Bran Powder (FFSBP)
**Moisture**	10.15 ^a^ ± 0.66	6.65 ^b^ ± 0.44
**Crude protein**	11.70 ^b^ ± 0.43	29.91 ^a^ ± 0.83
**Fat content**	0.85 ^b^ ± 0.08	2.00 ^a^ ± 0.07
**Crude fibers**	0.54 ^b^ ± 0.07	36.19 ^a^± 1.87
**Ash**	0.56 ^b^ ± 0.09	6.25 ^a^ ± 0.09
**Total carbohydrates**	86.35 ^a^ ± 1.72	25.65 ^b^ ± 0.87
**Energy value** (**kCal/100 g**)	401.93 ^a^ ± 0.60	312.62 ^b^ ± 0.54

Values are means ± SD of five determinations. Means within a row with different superscript letters (^a, b^) are significantly different (*p* < 0.05). Note: All components are expressed on a dry weight basis. Moisture is reported separately as a complementary parameter and is not included in the 100% sum of the dry matter components.

The reduction in carbohydrates is directly counterbalanced by a substantial increase in protein. The crude protein content of FFSBP (29.91%) is over 2.5 times greater than that of SWF (11.70%), confirming its role as a potent protein concentrate. This enhanced protein fraction is particularly valuable as the fermentation process may improve protein digestibility by producing smaller peptides and amino acids that are more easily absorbed [40]. Concurrently, a modest but statistically significant increase in fat content (from 0.85% in SWF to 2.00% in FFSBP) further contributes to the overall nutrient density of the bran powder [40]. This fat is likely attributable to residual sesame oil and lipids, and the fermentation process may enhance the profile of beneficial fatty acids, potentially improving health outcomes [40]. The incorporation of FFSBP introduces a profound quantitative shift in crude fiber and mineral content. The crude fiber content of FFSBP (36.19%) is extraordinarily high, being approximately 67 times greater than the negligible 0.54% found in SWF. This dramatic increase aligns with sesame bran’s identity as a lignocellulosic by-product and serves as a key functional attribute for fortification. Similar to wheat bran, another well-established source used to improve the nutritional profile of baked items, FFSBP’s high fiber content directly addresses the need to increase crude fiber in staple food products [41,42]. This positions FFSBP as a potent ingredient that can significantly boost the crude fiber content and functional properties of composite flour blends [43]. Beyond its fibrous matrix, the presence of unique lignans, which are retained in the bran, may provide additional health benefits such as cholesterol-lowering effects and protection against oxidative damage [43]. Furthermore, similar to the functional enhancement of wheat bran through bioprocessing, the prior fermentation of sesame bran likely contributes to its improved nutritional and functional properties [42]. Collectively, these attributes strongly support the potential of FFSBP as a functional ingredient capable of improving both the nutritional quality and consumer acceptability of fortified bakery products [43]. Similarly, the ash content, a proxy for total mineral matter, is over 11 times higher in FFSBP (6.25%) compared with SWF (0.56%). This substantial difference is consistent with the literature indicating that FFSBP is a rich source of essential minerals such as calcium, iron, magnesium, and zinc [44]. In stark contrast, the low mineral content in the refined wheat flour is a direct consequence of the milling process, which removes the mineral-rich bran and germ fractions [45]. Therefore, the incorporation of FFSBP effectively reintroduces these vital minerals into the food matrix. Furthermore, the lower moisture content of FFSBP (6.65%) compared with SWF (10.15%) reflects the efficacy of the freeze-drying process, which enhances the powder’s microbial stability and shelf-life, facilitating its storage and incorporation into composite flour blends.

The fermentation of sesame bran powder using *Lactobacillus plantarum* and subsequent freeze-drying significantly altered its chemical composition. The low total carbohydrate value (25.65%) in FFSBP aligns with the established principle that microbial fermentation reduces carbohydrate content as microbes consume simple sugars and starches [46,47]. While the high crude fiber content remained prominent, the fermentation process likely modified the fiber structure, potentially breaking down complex polymers and enhancing their bioavailability and functionality, as observed in fermented wheat bran where water-extractable arabinoxylans increase [46,48]. Concurrently, the process improves protein quality by increasing the abundance of bioactive peptides and free amino acids and enhancing protein solubility, as demonstrated in studies on fermented wheat germ and bran [46,49,50]. Furthermore, fermentation is known to increase antioxidant activity through the release of phenolic compounds and to degrade anti-nutritional factors like phytic acid, thereby enhancing the overall nutritional value and functional potential of the bran [46,47,48,50]. This biotransformation effectively upgrades FFSBP from a simple by-product into a functionally enhanced food ingredient.

In summary, the data unequivocally demonstrate that FFSBP is a nutritionally superior ingredient to refined wheat flour in terms of protein, fiber, and mineral density, while being lower in digestible carbohydrates and energy. Therefore, the partial substitution of SWF with FFSBP in burger bun formulations is a validated strategy to significantly enhance the protein, crude fiber, and mineral content of the final product, directly addressing the nutritional limitations of conventional refined wheat-based staples.

### 3.2. Techno-Functional Properties of Superior Wheat Flour (SWF), Fermented–Freeze-Dried Sesame Bran Powder (FFSBP) and Their Composite Flours

Table 3 shows the results of some techno-functional properties of wheat flour, Fermented–Freeze-Dried Sesame Bran Powder (FFSBP) and their composite flours. The most significant finding was the extraordinary functional capacity of pure FFSBP, which exhibited a Water Absorption Capacity (WAC) of 559% and an Oil Absorption Capacity (OAC) of 239%. These values are exceptionally high for a food ingredient and far exceed those of the control wheat flour (WAC: 147%, OAC: 149%). This indicates that the sequential fermentation and freeze-drying processes profoundly modified the physical structure of the sesame bran, likely creating a highly porous matrix with an extensive surface area that exposes a greater number of hydrophilic and lipophilic binding sites [51]. Consequently, the composite flours demonstrated a strong, dose-dependent increase in these absorption properties.

Notably, the integration of FFSBP maintained other key functional characteristics; the Emulsion Activity (EA) and Foam Capacity (FC) of the composite flours remained comparable to those of the full-wheat control, aligning with the principle that well-formulated composite flours can preserve the essential functional attributes of the base product [52]. However, while the incorporation of FFSBP offers significant functional and nutritional advantages, it is essential to consider that such modifications can influence dough rheology and final product texture. Therefore, adjustments in processing parameters, such as hydration time or mixing intensity, may be necessary to optimize the quality and consumer acceptability of the final baked products [53]. Consequently, the composite flours exhibited a strong, dose-dependent increase in both WAC and OAC. For instance, WAC increased from 154% at the 1% substitution level to 179% at the 5% level. This trend is directly attributable to the incremental addition of the highly absorptive FFSBP, which enhances the dough’s inherent ability to retain water and oil, a characteristic crucial for maintaining moisture, texture, and flavor in the final baked items [54,55]. In practical terms for bakery applications, this elevated WAC suggests that dough formulations with FFSBP may require adjusted hydration levels for optimal consistency. Similar to other fermented brans, FFSBP can improve the viscoelasticity and structural strength of the dough matrix, thereby enhancing the overall quality of the final product [56,57]. Simultaneously, the high OAC can improve mouthfeel and aid in the retention of fats and flavors within the baked bun. In contrast to the marked increases in absorption capacities, the Emulsion Activity (EA) and Foam Capacity (FC) of the composite flours exhibited notable stability. The EA of pure FFSBP (39.8%) was only marginally higher than that of SWF (37.1%), and all composite blends maintained EA values statistically similar to the control, ranging narrowly from 37.2% to 37.3%. This consistency suggests that the proteins in FFSBP, though modified by fermentation, retain effective emulsifying properties that are compatible with wheat gluten [58]. Similarly, the FC of FFSBP (18.0%) was slightly higher than that of SWF (15.1%), and the composite flours exhibited a very slight but consistent increase in FC with higher substitution levels. This indicates that the fermented bran powder does not disrupt and may slightly support the protein networks responsible for air cell formation and stabilization, a common challenge with high-fiber ingredients. The overall stability of both EA and FC across formulations indicates a versatile ingredient whose functional performance is robust [58,59]. This is a positive finding for product development, as it confirms that incorporating up to 5% FFSBP will not compromise the structural or textural qualities of batters and doughs that rely on these aerating mechanisms. The data demonstrate that Fermented–Freeze-Dried Sesame Bran Powder (FFSBP) is a functionally transformative ingredient. Its primary impact is the dramatic enhancement of the water- and oil-holding capacity of flour, a property that can be strategically leveraged to improve dough yield, final product softness, and shelf-life. These observed high absorption values align with the known techno-functional profile of sesame-based ingredients, which exhibit strong water- and oil-binding capabilities that can be further optimized at higher processing temperatures [59]. Crucially, this significant enhancement in hydration and fat retention was achieved without compromising the emulsifying and foaming functionalities critical to bakery product quality. This balance is noteworthy, as the incorporation of non-wheat components like sesame bran can alter dough rheology, often improving plasticity and reducing kneading time while potentially decreasing gluten content and elasticity [54]. However, the fermentation process of the bran confers counterbalancing benefits, enhancing the viscoelasticity and structural strength of the dough matrix [56,57]. As seen in the bioprocessing of wheat bran, fermentation can increase the solubility of dietary fibers like arabinoxylans and enhance enzyme activity, thereby improving the dough’s technological quality and the bread’s final volume and sensory acceptance [60,61,62]. Similarly, the fermentation of sesame bran in this study likely modified protein solubility and fiber structure, boosting functionality without destroying surface-active properties. Ultimately, the use of FFSBP aligns with the broader objective of nutritional fortification, as ingredients like sesame bran enhance the profile of baked products by contributing essential amino acids, unsaturated fatty acids, and minerals [54]. Furthermore, the fermentation step can increase the phenolic content and antioxidant activity of the final product [42]. Therefore, the supplementation with FFSBP successfully improves key functional properties critical for maintaining texture and freshness, while also elevating the nutritional value of the developed burger buns [62].

### 3.3. Chemical Composition and Physical Characteristics of Fortified Burger Buns

Table 4 presents the proximate composition and physical characteristics of the control and fortified wheat burger buns. The incorporation of Fermented–Freeze-Dried Sesame Bran Powder (FFSBP) induced significant (*p* < 0.05), dose-dependent alterations in both the nutritional profile and the baking performance of the final product.

#### 3.3.1. Moisture Content

The moisture content of the burger buns increased significantly (*p* < 0.05) with higher levels of FFSBP fortification, rising from 36.98% in the control formulation to 39.61% in the bun containing 5% FFSBP. This progressive trend is a direct technological consequence of the superior water-binding capacity inherent to the FFSBP ingredient (Table 3). FFSBP demonstrates an enhanced capacity to bind water, which is attributed to its high concentration of crude fiber and protein, both of which are intrinsically hydrophilic [58]. The improved moisture retention during dough preparation and baking is crucial for final product quality, as it contributes to a softer texture and enhanced freshness [54]. Furthermore, the incorporation of FFSBP modifies the dough’s structural–mechanical properties; while it may reduce the gluten network’s elasticity, it concurrently enhances dough plasticity, which can contribute to a more desirable crumb structure [54]. This mechanism aligns with observations from other fiber-rich ingredients, such as those derived from citrus and soy, which similarly improve water retention in baked items, underscoring the broader applicability of such functional components in baking science [63].

#### 3.3.2. Protein Content

The incorporation of Fermented–Freeze-Dried Sesame Bran Powder (FFSBP) into wheat flour significantly (*p* < 0.05) enhanced the protein content of the burger buns. As shown in Table 4, the crude protein level increased progressively from 12.78% in the control bun to 13.92% in the formulation with 5% FFSBP (95:5 blend). This enhancement is directly attributable to the high intrinsic protein concentration of FFSBP, which was determined to be 29.91% (Table 2), establishing it as an effective protein fortificant for wheat-based products [64]. Nutritionally, this incorporation is particularly valuable as it improves the profile of essential amino acids in the final product, notably lysine, which is typically the limiting amino acid in wheat [64]. From a technological perspective, the added protein from FFSBP alters the dough’s structural–mechanical properties. It can enhance dough plasticity, though it may concurrently dilute the wheat gluten content, potentially affecting fermentation dynamics and handling characteristics [54]. Despite these rheological changes, the judicious inclusion of FFSBP can contribute to favorable sensory attributes in the baked items, such as improved texture and flavor, which are critical for consumer acceptance [65]. However, it is important to note that, as with many fortificants, excessive substitution levels may lead to undesirable textural changes, underscoring the necessity for balanced formulations to optimize both nutritional gains and product quality [65].

#### 3.3.3. Fat Content

The fat content of the burger buns showed a slight but statistically significant (*p* < 0.05) increase with FFSBP fortification, rising from 4.25% in the control to 4.31% in the 95:5 formulation (Table 4). This modest increment corresponds directly to the higher native fat content of the FFSBP ingredient (2.00%) compared with the base wheat flour (0.85%) (Table 2). The consistent, progressive increase across the substitution gradient suggests the lipid components from FFSBP were evenly incorporated into the dough and effectively retained throughout the baking process [66]. Nutritionally, this contributes to a marginally richer fat profile, which may include beneficial fatty acids inherent to sesame, potentially enhancing the flavor and mouthfeel of the final product [67]. While the absolute increase is minor, it underscores the role of FFSBP in contributing to a denser nutritional matrix. However, as with all functional ingredient substitutions, maintaining a balance is crucial; excessive fat can negatively impact the desired texture and sensory qualities of leavened bakery [68]. The results indicate that at the incorporation levels studied (1–5%), FFSBP successfully enriched the product without destabilizing this balance.

#### 3.3.4. Crude Fiber Content

The most dramatic nutritional alteration observed was in the crude fiber content of the burger buns, which increased by over 238% from 0.76% in the control to 2.57% in the 95:5 FFSBP formulation (Table 4). This remarkable enrichment is the most definitive outcome of this study and stems directly from the exceptionally high fiber content of the FFSBP ingredient itself (36.19%) (Table 2). This result powerfully demonstrates the potential of valorizing this by-product to transform a low-fiber, refined wheat staple into a significant source of crude fiber. The efficacy of this fortification is amplified by the fermentation pre-treatment. The solid-state fermentation process not only elevates the crude fiber concentration but also modifies its structure, improving the solubility and fermentability of the crude fibers, which can enhance their nutritional impact and digestibility in the final food product [69]. Furthermore, fermentation enriches the bran with bioactive compounds, contributing additional health benefits such as antioxidant and antimicrobial properties [70,71]. This successful fiber enrichment model enables the incorporation of FFSBP into staple foods like bread and pasta, directly promoting higher crude fiber intake in the population [11,71]. Beyond nutrition, utilizing sesame bran represents a strategic approach to sustainable food systems, as it adds value to an agro-industrial by-product, thereby enhancing food quality while contributing to the reduction in processing waste [72].

#### 3.3.5. Ash Content

The ash content of the burger buns, which serves as a direct indicator of total mineral matter, increased significantly (*p* < 0.05) from 0.88% in the control to 1.17% in the 95:5 FFSBP formulation. This progressive trend is consistent with the literature indicating that FFSBP acts as a rich source of essential minerals, including calcium, magnesium, and iron [73,74]. By incorporating this ingredient, the study effectively reintroduces vital micronutrients that are substantially reduced during the refining of wheat flour, thereby enhancing the mineral density of the final product. This nutritional enhancement is particularly valuable for addressing micronutrient deficiencies in populations with diets high in refined grains [19]. Furthermore, the fermentation process applied to the sesame bran likely increases the bioavailability of these minerals by reducing antinutritional factors such as phytates, which can inhibit absorption [73]. Consequently, utilizing FFSBP in functional foods like fortified burger buns presents a viable strategy for improving dietary mineral intake and supporting overall health outcomes [75].

#### 3.3.6. Total Carbohydrates Content

The incorporation of Fermented–Freeze-Dried Sesame Bran Powder (FFSBP) into wheat burger buns significantly (*p* < 0.05) altered the macronutrient profile, most notably through a progressive reduction in total carbohydrate content. As detailed in Table 4, the carbohydrate level decreased from 81.33% in the control bun to 78.03% in the formulation containing 5% FFSBP. This reduction is a direct mathematical and nutritional consequence of the substitution effect, where nutrient-dense FFSBP rich in protein, fat, and especially crude fiber displaces a portion of the starchy wheat flour matrix [65]. Rather than diminishing the product’s value, this decrease in carbohydrates is concomitant with a substantial enhancement in overall nutritional density, as the buns are concurrently fortified with crude fiber, protein, and essential minerals [76]. From a dietary perspective, this shift in composition aligns with nutritional strategies for weight management, offering a food option with a lower available carbohydrate load for health-conscious consumers [77]. Furthermore, the increased fiber content and altered starch dynamics resulting from FFSBP inclusion suggest the potential for a moderated postprandial glycemic response, which could contribute to better blood sugar control [78]. Thus, the reduction in total carbohydrates signifies a favorable transformation towards a more nutrient-rich and functionally beneficial food product.

#### 3.3.7. Energy Values

The calculated energy value of the burger buns showed a small but statistically significant (*p* < 0.05) decrease with FFSBP fortification, declining from 416.21 kcal/100 g in the control to 410.51 kcal/100 g in the 95:5 formulation (Table 4). This reduction is a direct and expected outcome of the altered macronutrient composition, specifically the decrease in digestible carbohydrates and the increase in crude fiber, which contributes fewer calories per gram than starch or sugars. This shift results in a product with improved nutritional density, offering more protein and fiber per calorie consumed. The development of such high-fiber, lower-energy-density foods is beneficial for dietary strategies aimed at weight management and obesity prevention [77]. The significant influence of dietary fiber on overall energy density underscores its critical role in the design of health-focused diets [79]. Ultimately, the reduction in energy value is not a drawback but a positive indicator of successful nutritional fortification, reflecting a simultaneous boost in protein content and crude fiber, which enhances the overall nutritional profile and promotes satiety and digestive health [77].

#### 3.3.8. Physical Characteristics: Volume, Weight, and Specific Volume

The incorporation of FFSBP had a measurable and significant impact on the physical characteristics of the burger buns, revealing a critical technological trade-off between nutritional enhancement and product structure. A direct consequence of the superior water-binding capacity of FFSBP was a significant increase in the moisture content of the buns, which in turn contributed to a progressive and significant increase in final loaf weight across the formulations. However, this gain in weight contrasted with the behavior of the loaf volume. While the control bun achieved a volume of 217.10 cm^3^, the volume showed a declining trend, with significant reductions observed at the 4% and 5% substitution levels (Table 4). This inverse relationship is most critically captured by the specific volume (volume/weight ratio), a key quality index for leavened items. The specific volume decreased significantly, from 3.06 cm^3^/g in the control to 2.85 cm^3^/g in the 95:5 bun, a reduction of approximately 7% (Table 4). This decline underscores a common challenge in high-fiber baking: the enhancement of nutritional value through ingredients like FFSBP can adversely affect leavening quality and final product structure [80,81]. The insoluble fiber particles physically disrupt the continuous gluten network, weakening its elasticity and gas-retention capacity during proofing and oven spring, ultimately leading to a denser crumb. This inverse relationship, where increased nutritional benefits (such as fiber and antioxidants) come at the cost of reduced specific volume and potentially increased hardness, has been similarly observed in studies incorporating other bran or sesame flours [44,82]. Therefore, an optimization strategy is necessary. The data indicate that a substitution level of up to 3% FFSBP achieves substantial nutritional gains, nearly doubling the fiber content while maintaining physical properties statistically similar to the control. Levels of 4% and 5%, although delivering peak nutritional values, incur a significant cost in loaf volume and texture. This highlights the need to balance targeted nutritional goals with essential sensory and textural qualities for consumer acceptance. Future formulation work could explore avenues suggested by other studies, such as specific modifications to the bran or processing techniques, to enhance dough properties without compromising volume, thereby optimizing the balance between nutrition and quality [81,83].

### 3.4. Total Phenolics, Total Flavonoids and DPPH Radical Scavenging Activity % of Burger Buns Incorporated with Different Quantities of FFSBP

Table 5 shows total phenolics, total flavonoids and DPPH radical scavenging activity % of burger buns incorporated with different quantities of FFSBP. The total phenolic content (TPC) of the burger buns increased significantly (*p* < 0.05) in a dose-dependent manner with higher FFSBP substitution (Table 5), rising from 1.20 mg GAE/g dry weight in the control to 2.40 mg GAE/g in the 5% FFSBP bun, a 100% increase. This marked elevation results from the direct transfer of phenolic compounds from FFSBP into the food matrix. The solid-state fermentation of the bran using *Lactobacillus plantarum* was crucial to this enhancement. This bioprocess breaks down cell wall structures, liberating bound phenolic compounds and increasing their extractability and concentration in the final product [84,85]. Consequently, this process transforms a basic staple into a functional food with substantially greater antioxidant potential [86], contributing valuable bioactive compounds to dietary strategies aimed at mitigating oxidative stress [84,87]. These findings confirm that incorporating FFSBP can effectively elevate the bioactive profile and health-promoting potential of common bakery products.

The total flavonoid content (TFC) of the burger buns increased significantly (*p* < 0.05) and progressively with higher FFSBP substitution. The TFC rose from 0.27 mg CE/g dry weight in the control to 0.53 mg CE/g in the 95:5 formulation, representing an increase of approximately 96% (with intermediate values of 0.33, 0.38, 0.41, and 0.47 mg CE/g for the 99:1 to 96:4 blends, respectively) (Table 5). This marked enhancement is a direct result of incorporating flavonoid-rich FFSBP. The solid-state fermentation process using *Lactobacillus plantarum* likely played a key role, as microbial activity can hydrolyze conjugated flavonoid forms, thereby increasing the concentration of free, bioavailable compounds in the ingredient [88]. This bioprocess is known to enhance the antioxidant properties of plant-based materials [88]. The near-doubling of the flavonoid content signifies a critical functional upgrade, demonstrating that FFSBP fortification actively boosts specific health-relevant bioactive molecules. This elevates the buns from a basic staple to a functional food designed to deliver dietary antioxidants, positioning them as products with potential utility in dietary strategies aimed at preventing chronic diseases [89].

The DPPH radical scavenging activity of the burger buns increased significantly (*p* < 0.05) and in a pronounced dose-dependent manner with FFSBP fortification. The activity rose from 11.55 ± 0.63% in the control bun to 46.60 ± 2.45% in the 95:5 FFSBP formulation, representing a nearly fourfold enhancement (Table 5). This dramatic increase in antioxidant capacity is directly attributable to and strongly correlates with the concurrent, significant rise in total phenolic and flavonoid contents within the buns. These polyphenolic compounds, whose concentrations increased from 1.20 to 2.38 mg GAE/g and 0.27 to 0.48 mg CE/g, respectively (Table 5), are potent hydrogen donors that effectively neutralize stable free radicals like DPPH. The solid-state fermentation process applied to the sesame bran was instrumental, likely increasing the bioavailability and concentration of these bioactive antioxidants. This aligns with research showing that bakery products enriched with polyphenols and crude fibers can exhibit antioxidant activities more than double that of control samples [90]. Consequently, the incorporation of FFSBP successfully transformed the burger bun from a staple with minimal inherent antioxidant capacity into a functional food with substantial radical scavenging potential. This improved profile enhances the nutritional value and may provide additional health benefits, such as anti-inflammatory and cardioprotective effects, associated with these antioxidant compounds and the accompanying dietary fiber [91]. From a technological standpoint, the inherent antioxidant properties of FFSBP could also potentially extend the product’s shelf-life by mitigating oxidative rancidity [91].

### 3.5. Sensory Evaluation of Burger Buns Incorporated with Different Quantities of FFSBP

**Figure 1:**
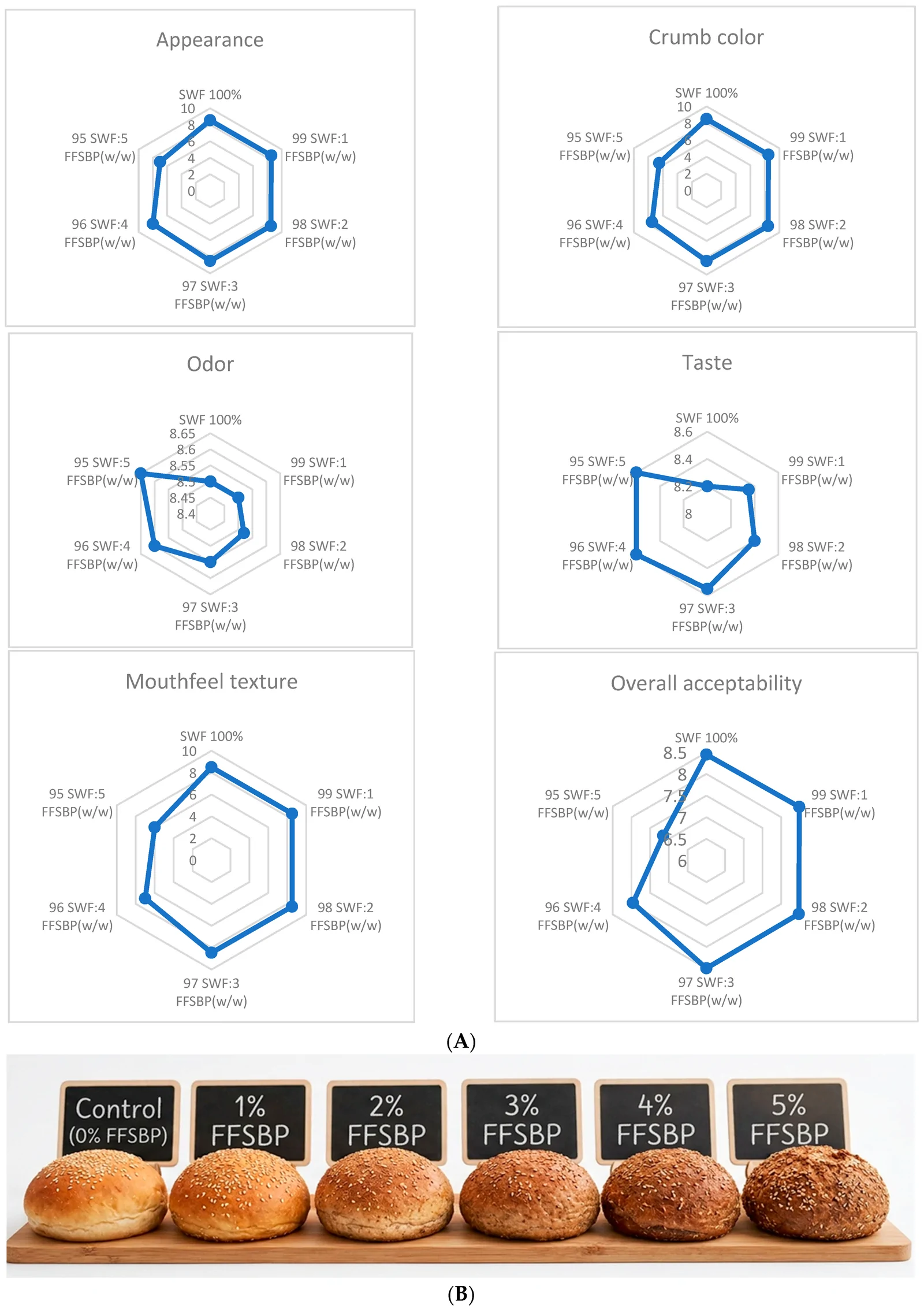
(**A**) Sensory evaluation of burger buns incorporated with different quantities of Fermented–Freeze-Dried Sesame Bran Powder (FFSBP). (**B**) Physical appearance of wheat flour burger buns incorporated with different quantities of FFSBP: (1) Control (0%), (2) 1%, (3) 2%, (4) 3%, (5) 4%, (6) 5% FFSBP.

**Appearance and Crumb Color**: Scores for appearance and crumb color remained stable and high (≈8.5) up to 3% FFSBP inclusion (Figure 1A). However, at higher substitution levels (4% and 5%), these scores decreased noticeably to 8.00/7.00 and 7.50/6.50, respectively (Figure 1A). This visual trend is clearly demonstrated in the physical comparison photographs (Figure 1B), which show the progressive darkening of both crust and crumb with increasing FFSBP levels. This suggests that FFSBP imparts a darker color, which may become less appealing to consumers at concentrations above 3%. The addition of FFSBP contributes to a darker crumb color, which may be perceived negatively by consumers at higher concentrations. Similar trends are observed with black sesame powder, where increased levels also lead to decreased lightness and overall acceptability beyond certain thresholds [82].

**Odor and Taste:** In contrast, odor and taste scores showed a positive trend with increasing FFSBP. Odor improved slightly from 8.50 (control) to 8.65 (5% FFSBP). Taste scores showed more pronounced improvement, rising from 8.20 (control) to a peak of 8.60 at 4–5% FFSBP (Figure 1). This indicates that FFSBP contributes desirable aromatic and flavor compounds, potentially nutty or fermented notes, which enhance these sensory attributes. This indicates that FFSBP contributes desirable aromatic and flavor compounds to the product matrix, potentially imparting nutty or roasted notes characteristic of sesame, which were favorably received by panelists. The marked improvement in taste scores suggests that FFSBP actively enriches the overall flavor profile of the buns. This finding aligns with other studies where the incorporation of sesame-derived ingredients improved the overall acceptability of baked goods such as cookies and biscuits [92].

**Mouthfeel Texture:** The mouthfeel texture of the burger buns was significantly influenced by FFSBP incorporation, revealing a clear threshold for acceptability. Sensory scores remained high and statistically similar to the control (8.50) at substitution levels up to 3%, indicating that low to moderate fortification preserved the desirable soft and cohesive crumb structure. However, a sharp and significant decline occurred at higher levels, with scores dropping to 7.10 at 4% and further to 6.00 at 5% FFSBP (Figure 1). This marked deterioration in texture is directly attributable to the composite effects of fortification: the dilution of vital gluten-forming proteins and the physical disruption of the dough network by insoluble crude fiber from the bran. This results in a denser, less elastic, and potentially drier crumb, a common technological challenge when incorporating high levels of bran into bread systems, as noted in studies with other bran types that negatively impact dough rheology and final bread quality [80]. Consistent with these findings, research on sesame cake incorporation has similarly reported harder crumb and reduced specific volumes at higher substitution levels [81]. This underscores a critical textural limitation for high-level FFSBP fortification, defining a practical upper bound for its application in quality leavened products.

**Overall Acceptability:** The calculated overall acceptability scores, derived from the mean of all sensory attributes, provide a comprehensive measure of consumer preference. The results demonstrate that formulations containing 1–3% FFSBP achieved overall acceptability scores (8.48–8.49) that were comparable to or slightly exceeded those of the control bun (8.45). This suggests that the enhancements in odor and taste provided by FFSBP at these levels successfully balanced the minor, non-significant changes in appearance, color, and texture, resulting in a product that was at least as liked as the traditional wheat flour bun. In contrast, the overall acceptability declined noticeably at 4% FFSBP (7.96) and more severely at 5% FFSBP (7.15). This decline clearly illustrates that the positive sensory contributions of FFSBP to flavor and aroma are outweighed by the negative impacts on visual appeal (appearance, color) and, most critically, mouthfeel texture at these higher concentrations. The sensory profile shifts from being flavor-enhanced to being perceived as visually less appealing and texturally inferior. The sensory evaluation results indicate that the optimal incorporation level for FFSBP in burger buns, from a sensory acceptability standpoint, lies within the 1% to 3% range. Within this range, it is possible to produce a nutritionally enriched bun with improved flavor without sacrificing overall consumer liking. Levels at or above 4% are not recommended due to significant compromises in texture and appearance that lead to a less acceptable final product.

### 3.6. Color Properties of Fortified Burger Buns

The color attributes of the burger buns, measured instrumentally for both crust and crumb, are presented in Table 6. The incorporation of Fermented–Freeze-Dried Sesame Bran Powder (FFSBP) induced significant (*p* ≤ 0.05) and dose-dependent alterations in the color profile of the final product, which aligns directly with the observed sensory trends for appearance and crumb color. The lightness (L*) of the bread crust decreased progressively and significantly with increasing FFSBP substitution, declining from 39.39 in the control to 37.65 in the 5% FFSBP formulation. Concurrently, the red/green coordinate (a*) increased from 14.60 to 15.80, and the yellow/blue coordinate (b*) increased from 17.50 to 19.60. This indicates that the crust became progressively darker, more reddish, and more yellowish with higher fortification levels. The darkening of the crust is a direct consequence of incorporating the inherently dark-colored sesame bran powder, which contains phenolic compounds, lignans, and tannins characteristic of sesame seed coats, as well as pigments from the seed coat layers [18,82]. Furthermore, the fermentation process may contribute to this effect by increasing the concentration of Maillard reaction precursors, such as reducing sugars and free amino acids, thereby enhancing non-enzymatic browning during baking [24,40]. The increase in b* (yellowness) is consistent with the typical hue associated with sesame products.

A similar, though less pronounced, trend was observed for the crumb. Crumb lightness (L) decreased significantly from 65.90 in the control to 64.83 in the 5% FFSBP bun. The a values, which were negative (indicating a slight greenish hue in the control wheat crumb), became less negative, moving from −0.55 towards zero (−0.32), signifying a reduction in greenness. The b* values (yellowness) increased significantly from 18.12 to 18.89. The darkening and shift in hue of the crumb are directly attributable to the physical incorporation of the darker FFSBP particles into the dough matrix. Similar results have been reported in studies incorporating black sesame powder [82] and other cereal brans into bread, where increased substitution levels consistently lead to decreased L* values [76,80]. This subtle shift in crumb color from the bright white of refined wheat to a creamier, slightly darker and more yellow hue is a common and expected outcome of fortification with bran materials.

These instrumental color data provide an objective basis for the sensory evaluation results, where panelists scored appearance and crumb color lower at substitution levels of 4% and 5% FFSBP. The significant darkening of both crust and crumb at these higher levels deviates from consumer expectations for conventional burger buns, which typically feature a golden-brown crust and a light, white crumb. While the color imparted by FFSBP at lower levels (1–3%) may be perceived as a desirable, “whole grain” characteristic, the pronounced darkening at 4–5% likely crosses a sensory threshold for many consumers, negatively impacting visual appeal [5,92].

From a technological and nutritional perspective, the observed color changes are not detrimental and may even be indicative of enhanced quality. The intensified browning could be associated with the increased levels of bioactive compounds and antioxidants contributed by the FFSBP, as phenolic compounds can participate in browning reactions [84,90]. However, the primary challenge lies in aligning these changes with consumer acceptance. Therefore, the color data strongly support the study’s overarching conclusion that an inclusion level of 3% FFSBP represents an optimal balance. At this level, the substantial nutritional and functional benefits are achieved while the associated modifications in crust and crumb color remain within a range that is acceptable to consumers, as evidenced by the maintained high sensory scores for appearance (≈8.5). This finding underscores the critical importance of integrating instrumental quality measurements with consumer perception studies when developing fortified staple foods with visually distinct ingredients such as sesame bran.

## 4. Conclusions

In conclusion, this study successfully demonstrated the potential of Fermented–Freeze-Dried Sesame Bran Powder (FFSBP) as a sustainable, functional ingredient for the nutritional enhancement of wheat flour burger buns. The fortification significantly and dose-dependently improved the nutritional profile, markedly increasing protein, crude fiber, mineral (ash) content, and enriching the buns with bioactive compounds such as phenolics and flavonoids. Consequently, the antioxidant activity of the buns was substantially elevated. However, these nutritional and bioactive gains were accompanied by technological trade-offs, notably a decrease in specific volume and a denser crumb texture at higher substitution levels, alongside a gradual darkening of crumb color. Sensory evaluation established a critical threshold for consumer acceptability. While fortification up to 3% FFSBP enhanced the taste and odor without significantly compromising overall acceptability, appearance, or texture, incorporation at 4% and 5% led to notable declines in sensory scores for mouthfeel and visual appeal. Therefore, an inclusion level of 3% FFSBP is recommended as optimal, achieving a substantial nutritional upgrade, particularly in fiber and protein, and a significant boost in antioxidant capacity, while maintaining sensory quality and acceptable physical characteristics comparable to the control. This research validates a practical strategy for transforming an underutilized agro-industrial by-product into a valuable fortificant, contributing to the development of healthier, functional staple foods that address common dietary nutrient gaps.

### Limitations of the Study

The authors acknowledge that the unfermented sesame bran powder was not included as a control in the experimental design, which would have allowed for a direct quantitative comparison of the effect of fermentation on the nutritional profile of sesame bran. While the observed improvements in protein, fiber, and mineral content are consistent with the well-documented effects of lactic acid fermentation on bran and oilseed by-products [20,21,46,47], future studies should include unfermented sesame bran as a control group to directly quantify the impact of fermentation. Additionally, future studies could further validate the sensory findings through instrumental texture profile analysis (TPA) to quantify hardness, adhesiveness, springiness, and other textural parameters, as well as electronic nose measurements to characterize the volatile odor profile of the fortified buns.

## Figures and Tables

**Table 1:** Formulation of wheat burger buns dough incorporating different quantities of Fermented–Freeze-Dried Sesame Bran Powder (FFSBP).

Ingredients	Wheat Flour 100% (Control)	SWF:FFSBP (*w*/*w*)				
		99:1	98:2	97:3	96:4	95:5
Wheat flour (g)	1000	990	980	970	960	950
Fermented–Freeze-Dried Sesame Bran Powder (FFSBP) (g)	-	10	20	30	40	50
Water (mL)	600	610	618	629.0	634.5	640.0
Dry active yeast (g)	12.5	12.5	12.5	12.5	12.5	12.5
White Sugar (g)	60	60	60	60	60	60
Salt (g)	10	10	10	10	10	10
Improver (g)	5.0	5.0	5.0	5.0	5.0	5.0
Sunflower oil (mL)	30	30	30	30	30	30

**Table 3:** Techno-functional properties of wheat flour, Fermented–Freeze-Dried Sesame Bran Powder (FFSBP) and composite flours.

Techno-Functional Properties	Superior Wheat Flour (SWF)	Fermented–Freeze-Dried Sesame Bran Powder (FFSBP)	SWF:FFSBP (*w*/*w*)				
			99:1	98:2	97:3	96:4	95:5
WAC (%)	147.00 ^g^ ± 1.89	559.00 ^a^ ± 2.42	154.02 ^f^ ± 1.90	159.84 ^e^ ± 1.96	167.36 ^d^ ± 1.77	171.48 ^c^ ± 1.99	178.60 ^b^ ± 2.02
OAC (%)	149.15 ^g^ ± 2.23	239.00 ^a^ ± 3.16	152.25 ^f^ ± 2.26	156.95 ^e^ ± 2.29	160.85 ^d^ ± 2.32	164.75 ^c^ ± 2.35	168.65 ^b^ ± 2.38
EA (%)	37.08 ^ab^ ± 1.83	39.8 ^a^ ± 1.20	37.15 ^ab^ ± 1.81	37.16 ^ab^ ± 1.12|	37.23 ^ab^ ± 1.44	37.28 ^ab^ ± 1.74	37.33 ^ab^ ± 1.82
FC (%),	15.08 ^b^ ± 0.66	18.03 ^a^ ± 0.62	15.13 ^b^ ± 0.60	15.17 ^b^ ± 0.44	15.21 ^b^ ± 0.33	15.25 ^b^ ± 0.36	15.28 ^b^ ± 0.61

Values are means ± SD of five determinations. Means in the same row with different letters are significantly different (*p* ≤ 0.05). Water absorption capacity (WAC), oil absorption capacity (OAC), emulsion activity (EA), and foam capacity (FC).

**Table 4:** Chemical composition and physical characteristics of burger buns incorporated with different quantities of Fermented–Freeze-Dried Sesame Bran Powder (FFSBP).

Components (g/100 g DW)	SWF 100%	SWF:FFSBP (*w*/*w*)				
		99:1	98:2	97:3	96:4	95:5
Moisture	36.98 ^f^ ± 1.45	37.52 ^e^ ± 1.14	38.11 ^d^ ± 1.44	38.58 ^c^ ± 0.78	39.18 ^b^ ± 0.89	39.61 ^a^ ± 1.48
Protein	12.78 ^e^ ± 0.88	13.02 ^de^ ± 0.94	13.22 ^d^ ± 063	13.47 ^c^ ± 0.58	13.69 ^b^ ± 0.33	13.92 ^a^ ± 0.29
Fat	4.25 ^c^ ± 0.32	4.26 ^bc^ ± 0.25	4.28 ^b^ ± 0.32	4.29 ^a^ ± 0.27	4.30 ^a^ ± 0.28	4.31 ^a^ ± 0.13
Crude fiber	0.76 ^f^ ± 0.08	1.11 ^e^ ± 0.09	1.46 ^d^ ± 0.08	1.82 ^c^ ± 0.09	2.20 ^b^ ± 0.06	2.57 ^a^ ± 0.07
Ash	0.88 ^e^ ± 0.04	0.96 ± 0.04	1.04 ± 0.04	1.06 ± 0.04	1.12 ± 0.04	1.17 ± 0.04
Total carbohydrates	81.33 ^a^ ± 0.33	80.65 ^ab^ ± 0.36	80.00 ^b^ ±0.27	79.36 ^bc^ ±0.25	78.69 ^c^ ± 0.20	78.03 ^d^ ± 0.14
Energy value (kCal/100 g)	416.21 ^a^ ± 0.32	415.26 ^b^ ± 0.27	414.20 ^b^ ± 0.22	413.11 ^c^ ± 0.17	411.85 ^cd^ ± 0.12	410.51 ^d^ ± 0.11
Volume (cm^3^)	217.10 ^a^ ± 2.23	216.08 ^a^ ± 2.11	216.26 ^a^ ± 2.19	215.30 ^ab^ ± 2.10	213.58 ^b^ ± 2.09	206.79 ^c^ ± 1.30
Loaf weight (g)	70.95 ^c^ ± 0.33	71.55 ^b^± 0.72	71.85 ^b^ ± 0.41	72.10 ^ab^ ± 0.60	72.40 ^a^± 0.48	72.56 ^a^ ± 0.23
Specific volume (cm^3^ g^−1^)	3.06 ^a^ ± 0.08	3.02 ^a^± 0.08	3.01 ^a^ ± 0.09	3.00 ^ab^ ± 0.10	2.95 ^b^ ± 0.11	2.85 ^c^ ± 0.13

Values are means ± SD of five determinations. Means in the same row with different letters are significantly different (*p* ≤ 0.05). SWF 100%: control (100% wheat flour). FFSBP: Fermented–Freeze-Dried Sesame Bran Powder. Note: All components are expressed on a dry weight basis. Moisture is reported separately as a complementary parameter and is not included in the 100% sum of the dry matter components.

**Table 5:** Total phenolics, total flavonoids, and DPPH radical scavenging activity % of burger buns incorporated with different quantities of Fermented–Freeze-Dried Sesame Bran Powder (FFSBP).

Parameter	SWF 100%	SWF:FFSBP (*w*/*w*)				
		99:1	98:2	97:3	96:4	95:5
TP	1.20 ^e^ ± 0.09	1.45 ^d^ ± 0.08	1.67 ^c^ ± 0.10	1.91 ^cb^ ± 0.11	2.18 ^b^ ± 0.13	2.40 ^a^ ± 0.12
TF	0.27 ^d^ ± 0.02	0.33 ^c^ ± 0.02	0.38 ^bc^ ± 0.02	0.41 ^b^ ± 0.03	0.47 ^ab^ ± 0.07	0.53 ^a^ ± 0.06
DPPH %	11.55 ^f^ ± 0.63	18.96 ^e^ ± 1.05	26.53 ^d^ ± 1.40	33.92 ^c^± 1.75	39.99 ^b^ ± 2.10	46.60 ^a^ ± 2.45

Values are means ± standard deviation (SD) of five determinations. Values followed by the same letter are not significantly different (*p* < 0.05). TP = total phenolics (mg gallic acid/g dry weight); TF = total flavonoids (mg catechin equivalents (CE)/g dry weight).

**Table 6:** Color properties of burger buns incorporated with different quantities of Fermented–Freeze-Dried Sesame Bran Powder (FFSBP).

	SWF 100%	SWF:FFSBP (*w*/*w*)				
		99:1	98:2	97:3	96:4	95:5
**Bread crust color**						
L*	39.39 ^a^ ± 0.46	39.05 ^b^ ± 0.42	38.70 ^c^ ± 0.32	38.25 ^d^ ± 0.38	37.95 ^e^ ± 0.39	37.65 ^f^ ± 0.34
a*	14.60 ^f^ ± 0.30	14.75 ^e^ ± 0.28	14.90 ^d^ ± 0.26	15.10 ^c^ ± 0.28	15.45 ^b^ ± 0.24	15.80 ^a^ ± 0.22
b*	17.50 ^f^ ± 0.18	17.75 ^e^ ± 0.24	18.00 ^d^ ± 0.23	18.30 ^c^ ± 0.22	18.85 ^b^ ± 0.21	19.60 ^a^ ± 0.20
**Bread crumb color**						
L*	65.90 ^a^ ± 0.50	65.60 ^b^ ± 0.48	65.30 ^c^ ± 0.46	65.00 ^d^ ± 0.45	64.90 ^e^ ± 0.44	64.83 ^f^ ± 0.42
a*	−0.55 ^a^ ± 0.05	−0.50 ^b^ ± 0.05	−0.45 ^c^ ± 0.04	−0.40 ^d^ ± 0.04	−0.35 ^e^ ± 0.03	−0.32 ^f^ ± 0.04
b*	18.12 ^g^	18.30 ^e^ ± 0.14	18.45 ^d^ ± 0.18	18.60 ^c^ ± 0.12	18.75 ^b^ ± 0.16	18.89 ^a^ ± 0.15

Values are means ± SD of five determinations. Means in the same row with different letters are significantly different (*p* ≤ 0.05).

## Data Availability

The original contributions presented in this study are included in the article. Further inquiries can be directed to the corresponding author.
